# An Antibody-Aptamer-Hybrid Lateral Flow Assay for Detection of CXCL9 in Antibody-Mediated Rejection after Kidney Transplantation

**DOI:** 10.3390/diagnostics12020308

**Published:** 2022-01-25

**Authors:** Lisa K. Seiler, Ngoc Linh Phung, Christoph Nikolin, Stephan Immenschuh, Christian Erck, Jessica Kaufeld, Hermann Haller, Christine S. Falk, Rebecca Jonczyk, Patrick Lindner, Stefanie Thoms, Julia Siegl, Günter Mayer, Regina Feederle, Cornelia A. Blume

**Affiliations:** 1 Institute of Technical Chemistry, Leibniz University Hannover, 30167 Hannover, Germany; seiler@iftc.uni-hannover.de (L.K.S.); phung@iftc.uni-hannover.de (N.L.P.); rjonczyk@iftc.uni-hannover.de (R.J.); lindner@iftc.uni-hannover.de (P.L.); thoms@iftc.uni-hannover.de (S.T.); 2 Institute of Transfusion Medicine and Transplant Engineering, Hannover Medical School, 30625 Hannover, Germany; nikolin.christoph@mh-hannover.de (C.N.); immenschuh.stephan@mh-hannover.de (S.I.); 3 Helmholtz Centre for Infection Research, Cellular Proteome Research Group, 38124 Braunschweig, Germany; christian.erck@helmholtz-hzi.de; 4 Department of Nephrology and Hypertension, Hannover Medical School, 30625 Hannover, Germany; kaufeld.jessica@mh-hannover.de (J.K.); haller.hermann@mh-hannover.de (H.H.); 5 Institute for Transplant Immunology, Hannover Medical School, 30625 Hannover, Germany; falk.christine@mh-hannover.de; 6 Chemical Biology & Chemical Genetics, Life and Medical Sciences (LIMES) Institute, University of Bonn, 53121 Bonn, Germany; jsiegl@uni-bonn.de (J.S.); gmayer@uni-bonn.de (G.M.); 7 Center of Aptamer Research & Development (CARD), University of Bonn, 53121 Bonn, Germany; 8 Monoclonal Antibody Core Facility, Institute for Diabetes and Obesity, Helmholtz-Zentrum München, German Research Center for Environmental Health, 85764 Neuherberg, Germany; regina.feederle@helmholtz-muenchen.de

**Keywords:** biomarkers, antibody-mediated rejection (AMR), neural net analysis, CXCL9 (MIG), aptamer, antibody, aptamer-antibody-hybrid lateral flow assay (hybrid-LFA)

## Abstract

Chronic antibody-mediated rejection (AMR) is a key limiting factor for the clinical outcome of a kidney transplantation (Ktx), where early diagnosis and therapeutic intervention is needed. This study describes the identification of the biomarker CXC-motif chemokine ligand (CXCL) 9 as an indicator for AMR and presents a new aptamer-antibody-hybrid lateral flow assay (hybrid-LFA) for detection in urine. Biomarker evaluation included two independent cohorts of kidney transplant recipients (KTRs) from a protocol biopsy program and used subgroup comparisons according to BANFF-classifications. Plasma, urine and biopsy lysate samples were analyzed with a Luminex-based multiplex assay. The CXCL9-specific hybrid-LFA was developed based upon a specific rat antibody immobilized on a nitrocellulose-membrane and the coupling of a CXCL9-binding aptamer to gold nanoparticles. LFA performance was assessed according to receiver operating characteristic (ROC) analysis. Among 15 high-scored biomarkers according to a neural network analysis, significantly higher levels of CXCL9 were found in plasma and urine and biopsy lysates of KTRs with biopsy-proven AMR. The newly developed hybrid-LFA reached a sensitivity and specificity of 71% and an AUC of 0.79 for CXCL9. This point-of-care-test (POCT) improves early diagnosis-making in AMR after Ktx, especially in KTRs with undetermined status of donor-specific HLA-antibodies.

## 1. Introduction

Kidney transplantation (Ktx) is the most frequent transplantation worldwide, but transplant survival is limited by a high incidence of late antibody-mediated rejection (AMR) [1,2]. KTRs are regularly and continuously monitored by detection of donor-specific antibodies (DSAs) and transplant biopsies.

Mechanisms and causes of AMR have been investigated by plasma cell and biopsy analyses [1,3,4]. Identification of DSAs along with several biopsy-based morphological and molecular criteria are relevant for AMR diagnosis according to the current BANFF-classification criteria [5]. Although DSAs are important, they are not an unambiguous, diagnostic criterion for AMR [5]. Specifically, detection of de novo DSAs after Ktx can lead to perform protocol biopsies [6]. However, it has been shown that circulating DSAs in KTRs with an inconspicuous clinical course do not necessarily indicate ongoing rejection of the graft, and in some cases with a histological finding suspicious for AMR, single antigen HLA-DSA cannot be identified by solid phase assays (SPA) and therefore new methods for HLA antibody screening were built up [7,8]. This has also been demonstrated in a recent cross-sectional analysis of stable recipients with DSA-positivity 6 months post Ktx, of which only 51% were diagnosed with biopsy-proven AMR [9]. Despite detailed guidelines for the practical use of DSA detection with the modern sensitive but costly SPA methods, interpretation of the results is complex and the diagnostic value as post-transplant monitoring is considered to be limited [10,11]. C4d deposition of peritubular capillaries in transplant biopsies were also not consistently associated with the full histopathological finding of AMR [12,13]. Thus, an alternative easy and cheap diagnostic for recognition of AMR is urgently needed based upon biomarkers with high specificity for AMR [14,15].

Consequently, numerous approaches for non-invasive, non-DSA-based diagnostic strategies have been explored in recent years. Among the diverse immunological biomarkers that could be relevant, many non-invasive biomarkers have shown potential in preliminary studies. The chemokines CCL3, CCL4, CXCL9, CXCL10 and CXCL11 have recently been identified as markers for inflammation of the microvascular blood vessels [16,17,18,19,20,21,22] and chemokines play a role as early markers in renal allograft rejection [15,23,24]. They act as a signaling system between allogen-presenting cells such as macrophages and immunocytes. In addition, cytokines can be produced by activated graft endothelium as part of the organ-related rejection process [25,26] with low-level activation of T-cells, natural killer (NK) cells and myeloid cells. Cellular margination and activation including monocytes or macrophages and NK cells was described in AMR in parallel to an altered proinflammatory gene expression profile, also in biopsies with a classification to be unsuspicious [27].

Translation of these biomarkers to the clinic requires robust validation studies and standardized, commercially available assays that need to be paralleled, but not solely depending on increased serum creatinine levels [28], which may also indicate AMR. Test platforms for reliable detection of appropriate rejection markers usually require expertise due to their complex handling and interpretation, including additional equipment, and, thus, are not applicable for POCT. Hence, an LFA might serve as an alternative approach, because the patient is able to perform the test at home and to recognize the test result by eye within minutes. 

Such sandwich assays usually apply antibodies, but new test setups are being sought in the field of LFAs as the use of antibodies has some disadvantages and limitations: commonly generated in animals or cells, batch-to-batch variations, limited shelf life, difficult to label at a specific location and sensitive to temperature, possibly even irreversible denatured. In comparison, aptamers, which are single-stranded DNAs or RNAs, can be chemically synthesized [29], modified with functional groups, in most cases without loss of affinity [30]. Furthermore, they are thermally stable, cheap to produce, can detect small molecules and provide a constant production quality. Initial aptamer-based sandwich tests have already been described [31,32,33,34,35] and various LFAs have been reported using a hybrid of aptamer and antibodies for the composition of the sandwich [36,37,38].

It was the aim of this study to select AMR biomarkers and to investigate their diagnostic potential at the day of biopsy to develop an LFA for straight forward and early detection in urine of patient with AMR after Ktx.

## 2. Materials and Methods

### 2.1. Patient Samples, Data and Ethic Vote

Patient samples and data were derived from two independent cohorts of KTRs from a protocol biopsy program, collected within a defined biomarker study (Ethic approval Hannover Medical School, MHH No. 9682011 and MHH No. 7370) at the day of a biopsy. The 1st cohort included 120 KTRs, aged between 18 and 75 years, transplanted between 1990 and 2012; the 2nd cohort included 78 partially pediatric KTRs, aged between 5 and 75 years, transplanted between 1986 and 2017. Patients of the 1st cohort had a relatively decreased transplant age as compared to patients of the 2nd cohort. In addition, patients of the 2nd cohort more often had a tacrolimus/mycophenolate/steroid-based immunosuppressive regimen (classical tacrolimus-triple-therapy). In patients with repeated biopsies, biopsies as well as urine and serum samples were analyzed only in case of a histological diagnosis of rejection. Eight sets of data out of the 1st cohort were excluded for biomarker determination due to diagnoses other than rejection (glomerulonephritis, diabetic nephropathy, polyomavirus nephropathy). Whole-urine samples used were free of leukocytes or macro-proteinuria. Kidney graft function and further rejection episodes were followed up for 4.5 years in the 1st and for 9 months in the 2nd patient cohort. Identification of single DSAs in AMR was performed ±3 months around the biopsy (One Lamda, Canoga Park, CA, USA) as described before [39]. Anti-HLA antibodies were detected using LABScreen-Mixed Assays (One Lamda, Canoga Park, CA, USA). All samples of the 1st patient cohort with positivity for HLA class I or II and an MFI value >1000 underwent the LABScreen Single antigen test (One Lamda) according to the manufacturer’s guidelines. HLA-specific antibodies were assigned as DSA against the grafted kidney according to the HLA-A-, B, -C, -DR, DPB- and DQ typing of donor and KTRs. Biopsies were analyzed using the BANFF-classification that was applicable at the time of biopsy [40,41].

### 2.2. Determination of Biomarkers

Chemokines, cytokines or growth factors as possible biomarkers must be stable enough to be detected in patients’ urine despite ongoing immunosuppression comprising different individual drug regimens and individual immunity conditions. Therefore, we chose an approach with a comprehensive panel of 65 markers in different sample cohorts (cohorts of patients with TCMR or AMR or a biopsy finding unsuspicious for any rejection). The markers were comparatively tested using a Luminex-based multiplex assay in plasma of patients from the 1st cohort, comprising 16 KTRs with AMR. A selected panel of 14 markers (Supplementary Table S1) was tested in plasma and urine samples from patients of the 2nd cohort including 23 partially pediatric KTRs with AMR. Immediately after collecting, EDTA blood samples were processed by centrifugation and plasma and urine aliquots were stored at −80 °C. Biomarker levels were quantified according to the manufacturer’s instructions (BioRad, Hercules, CA, USA), as described previously [39,42,43]. In addition to histopathological scoring samples, 44 second biopsy samples were taken in the 1st patient cohort with written informed consent approved by the ethics committee of MHH. Biopsies were snap-frozen and manually dissected to separate capsule, cortex and medulla regions. Protein lysates of cortex sections were generated by Bio-Plex Cell Lysis Kit (Bio-Rad, Hercules, CA, USA) with adjustment of the total protein concentration to 12.5 µg per sample.

### 2.3. Statistics

Using the method of neural net analysis, 65 biomarkers and four clinical parameters (age at Ktx, time between biopsy and Ktx, gender, serum creatinine) were scored. As the clinical parameters serve as confounders, markers that are clinically relevant for rejection were involved (older kidney grafts more often develop an AMR, serum creatinine is an important marker for rejection and an age- and gender-caused tendency for rejection should be ruled out). Two-layer feed-forward networks with 5–20 neurons in the hidden layer were used. In total, 250 feature selection runs were carried out per neuron. A ranking order for the various markers was calculated by evaluation of score values. For this classification tasks with a given set of classes, supervised learning schemes were used. A set of training samples contained input values together with the desired output. In combination with an error function, the training set was used to adjust the internal parameters of the network. For classification, this function mapped the values of the parameters to ’rejection´ or ´non-rejection´, while ’rejection’ contained values of all rejection subgroups (T-cell mediated rejection (TCMR), borderline-TCMR and AMR). 

Among the best-scored 15 biomarkers, biomarker values of all rejection subgroups were compared using Wilcoxon test versus the KTR group with non-rejection, the level of statistical significance was indicated by asterisks. ROC analyses were performed to display the area under the curve (AUC) as well as the sensitivity and specificity of the LFA as compared to eGFR at the day of biopsy.

### 2.4. Generation and Selection of Monoclonal Antibody against CXCL9

Antibody generation was performed by Helmholtz Munich (HMGU, Germany) based upon standardized immunization of rats with commercial CXCL9 (OriGene, MD, America) and using the mouse myeloma cell line P3X63-Ag8.653 (ATCC CRL-1580) as described elsewhere [44]. 19 rat hybridoma cell supernatants were transferred to a self-designed sandwich test to evaluate CXCL9 binding properties and affinities of the antibody preparations. Therefore, wells of a high-binding microtiter plate were coated with 50 µL per well in carbonate buffer pH 9.6 donkey anti-rat (1:1000, Jackson Immuno-Research, United Kingdom) and incubated overnight before blocking with 200 µL per well blocking buffer (1 h). Undiluted hybridoma supernatant (100 µL) was incubated with self-made CXCL9 (100 μL, 35 ng∙mL^−1^) as a positive control for 1 h and secondly with a range of CXCL9 of different concentrations (0 to 20 ng∙mL^−1^). Supernatant with self-made CXCL9 used was produced by γ-interferon-stimulated human macrophages according to the method by Sudan et al. [45]. The ELISA was built up by sequential addition of CXCL9 specific antibody (MIG DuoSet ELISA, Cy392-05, R&D Systems; 100 µL, 1 h), biotinylated antibody (Biotin Anti-LC-kappa Conjugate, Thermo Fisher, 100 µL, 0.5 h) and peroxidase with streptavidin (100 µL, 0.5 h). Between all incubations steps (RT), supernatant was removed and wells were washed three times with PBS. Signalling was quantified upon absorption at 450 nm (Sunrise absorption microplate reader, Tecan, Swiss) using the dye TMB (Kementec, Taastrup, Denmark; 5 min, 50 μL) after addition of sulfuric acid (1 M, 100 μL) as stopping reactant. Finally, LFA experiments in this study were performed with purified antibody clone CXCL9 15A5 (rat IgG2b) as capture antibody.

### 2.5. Aptamer Sequence

The sequences of applied ssDNAs and their modifications are summarized in Table 1. The aptamer G123 was identified by Systematic Evolution of Ligands by Exponential Enrichment (SELEX). Briefly, the aptamer consists of 84 nucleotides including primer regions with a length of 21 nucleotides and shows a binding affinity to CXCL9 with an equilibrium dissociation constant (KD) of 92 ± 14 nM (details of the selection and characterization of G123 binding to CXCL9 will be published in a separate manuscript elsewhere). G123 was modified with a disulfide linker at the 5′-terminus and extended with a hexaethylene glycol spacer: C6-SS-HEG-5′-G123-3.

### 2.6. Further Components of the LFA

Nitrocellulose membrane Unisart^®^ CN95 was kindly provided by Sartorius Stedim Biotech (Goettingen, Germany). Gold nanoparticles (AuNP) were kindly provided by Fassisi GmbH (Goettingen, Germany). Commercial CXCL9 (Cat# TP720369) was acquired from OriGene (MD, USA). Trehalose was purchased from Fluka BioChemika (Steinheim, Germany). Sucrose and bovine serum albumin (BSA) were obtained from Sigma-Aldrich GmbH (Munich, Germany). Tween20 was purchased from PanReac AppliChem (Darmstadt, Germany). Phosphate buffered saline (PBS, pH 7.4) was prepared (supplement, methods). Blocking buffer was based upon PBS supplemented with 10% FCS (Sigma-Aldrich Chemie GmbH, Taufkirchen, Germany). PBS was prepared with 137 mM sodium chloride, 2.7 mM calcium chloride, 10.1 mM disodium hydrogen phosphate and 1.8 mM calcium hydrogenphosphate (all purchased from Sigma-Aldrich GmbH, Munich, Germany). Solution pH was adjusted to 7.4. Sodium borate buffer was prepared with 2 mM sodium tetraborate (Sigma–Aldrich Chemie GmbH, Taufkirchen, Germany) and set to pH 9. Sodium borate buffer was prepared with 2 mM sodium tetraborate (Sigma–Aldrich Chemie GmbH, Taufkirchen, Germany). Binding buffer (BB) is PBS based and contained 138 mM sodium chloride, 5 mM potassium chloride, 8.1 mM disodium hydrogen phosphate and 1.5 mM potassium dihydrogen phosphate, 1 mM magnesium chloride, 1 mM calcium chloride, 170 mM urea, 7 mM ammonium acetate. BB (pH 6.5) was PBS-based with its content adjusted to urine milieu, supplemented with 0.25% BSA (Sigma-Aldrich GmbH), 0.1 mg∙ml^−1^ salmon sperm DNA (Thermo Fisher, MA, USA) (supplement, methods).

### 2.7. Preparation of Gold Nanoparticle-Aptamer Conjugates (AuNP-G123)

Preparation of AuNP-G123 was performed according to an adjusted protocol of Phung et al. [46]. Conjugates were analyzed by dynamic light scattering (DLS, Particle Analyzer LitesizerTM 500, Anton Paar, Graz, Austria) and UV-Vis (Epoch Mikroplatten-Spektralphotometer, BioTek Instruments, Winooski, USA).

### 2.8. Preparation of Test Strips for the LFA

The hybrid-LFA was composed of a sample pad (Åhlstrom-Munsjö, Helsinki, Finland) and an absorbent pad (Åhlstrom-Munsjö, Helsinki, Finland, grade 222) glued to a backing card (GE-Whatman, 7.5 cm) with 2 mm overlap to the centered nitrocellulose membrane. Sample pads were incubated for 2 h with 100 mM Tris (pH 8.0) supplemented with 1% BSA, 1% sucrose and 0.05% Tween 20 and dried at RT overnight before they were applied on the backing cards. Solutions for test (A, 1 mg∙ml^−1^) and control zone were automatically applied onto the nitrocellulose membrane (Fassisi GmbH, Goettingen, Germany, 1 µL∙cm^−1^), in some cases by hand (0.2 µL). Beforehand, streptavidin (20 µM in PBS, Roth, Germany) and the same volume of biotinylated oligonucleotide solution (20 µM in PBS) was mixed and incubated for 2 h at 500 rpm to prepare the solution for the control zone. Test and control zones were dried for 3 h at 50 °C, cut into strips of 4 mm and placed in an airtight bag under exclusion of light.

### 2.9. LFA Evaluation with Technical and Spiked Samples

In total, 100 µL of each sample (technical sample, spiked sample or patient sample) were placed at RT in a well of a 96-well plate. A total of 1 µL AuNP-G123 was applied onto the conjugate pad. Concentrations of purified CXCL9 were diluted in BB (technical sample) or in BB combined with pooled patient negative urine samples (spiked samples). Pooled urine samples derived of four KTRs with non-rejection proven by biopsy. All patient samples were diluted in BB (ratio 1:1). LFA strips were placed in a sample well and scanned (Epson V370) after 15, 30 and 45 min before quantitative analysis by eye as well as by ImageJ-estimation of scans (ImageJ 1.46r, NIH, USA).

## 3. Results

### 3.1. Patients

#### 3.1.1. Characteristics of the Two Independent Patient Cohorts

Two independent patient cohorts were included in this work. The 1st cohort, recruited between 2011 and 2012, served for biomarker selection (see methods) and here 112 data sets, plasma samples of 112 KTRs and 2nd biopsies of 44 KTRs out of these group were available. There was a slightly higher percentage of protocol biopsies (55.4%) in the 1st cohort. According to the BANFF-classification at that time [41], biopsy findings showed in 46.4% the diagnosis ‘non-rejection’, in 27.7% the diagnosis ‘borderline-TCMR’, in 11.6% the diagnosis TCMR and in 14.3% the diagnosis AMR (at that time the presence of HLA-antibodies was not obligatory for this diagnosis). A major part of the 78 patients of the 2nd patient cohort, recruited in 2017 and 2018, received for cause biopsies (65.4%). Biopsy findings here were classified according to the revisited BANFF-classification [5,40] with the diagnoses ‘non-rejection’ in 51.3%, borderline-TCMR in 10.3%, TCMR only in 8.9% and AMR in 29.5% (Supplementary Table S2). The mean transplant age was higher in the 2nd cohort and the 2nd cohort included 12 children.

#### 3.1.2. Immunosuppression and Graft Function

Immunosuppressive therapies were frequently minimized or calcineurin-inhibitors were replaced by an mTOR-inhibitor in AMR and borderline-TCMR-patients of the 1st cohort as well as in 50% of patients with borderline-TCMR and TCMR and ~30% of patients with AMR of the 2nd cohort. 12 children, who were included in the 2nd cohort, received steroid-sparing therapy (Supplementary Tables S3 and S4).

**Figure 1:**
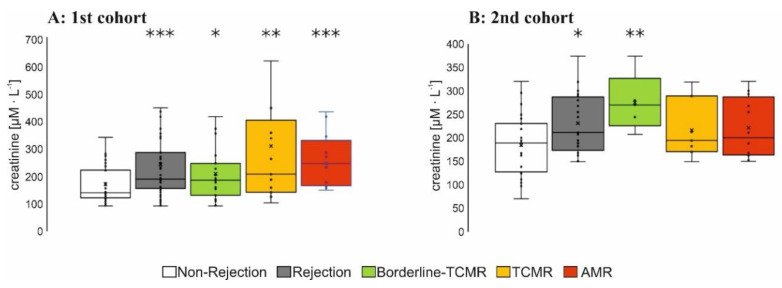
Serum creatinine of 1st (**A**) and 2nd patient cohort (**B**): Serum Creatinine was determined at the day of the protocol or for-cause-biopsy in kidney transplant recipients with non-rejection, borderline-TCMR, TCMR (T-cell mediated rejection) or AMR (antibody-mediated rejection) as indicated. Boxplots show the median value, the mean and the standard error of the mean. Wilcoxon test revealed a statistical significance between groups with * *p* < 0.05; ** *p* < 0.01; *** *p* < 0.001 as indicated.

Serum creatinine was significantly higher in all groups with graft rejection of the 1st cohort (Figure 1A), possibly due to a higher proportion of cyclosporine-treated patients in this group. In total, 16 AMR-patients of the 1st cohort exhibited AMR with transplant loss within 4.5 years. In total, 17 of 23 AMR-patients in the 2nd cohort, followed up for 9 months after the first biopsy, had further biopsies with AMR in a functioning graft (Supplementary Tables S5 and S6).

#### 3.1.3. Panel-Reactive Antibodies (PRA) and DSAs

A general screening for HLA I or II DSAs was positive in all AMR-patients of the 1st and 2nd cohort (Supplementary Tables S7 and S8). At the time of biopsy ± 3 months, defined HLA-DSAs according to SPA were identified in one patient with borderline-TCMR and in 5 out of 16 patients with AMR (Supplementary Table S5) in the 1st patient cohort and in 11 out of 23 AMR-patients in the 2nd patient cohort (Supplementary Table S6). Median PRAs for AMR were relatively low (1st patient cohort (median/range): AMR 29%, 2–70%, 2nd cohort: AMR: 23%, 0–100).

### 3.2. Biomarkers

#### 3.2.1. Plasma Level of Relevant Biomarkers in Patients with AMR

**Figure 2:**
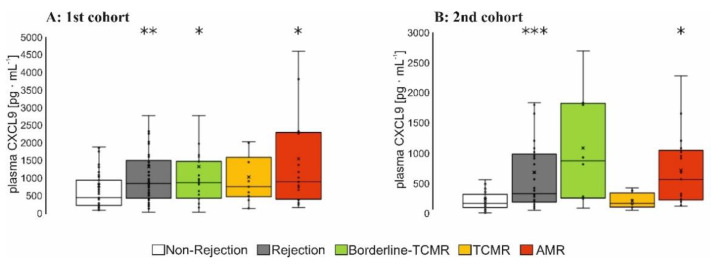
The 1st (**A**) and 2nd patient cohort (**B**): CXCL9 was determined using the Luminex-based multiplex protein array in the cryo-preserved EDTA-processed plasma samples of the kidney transplant recipients at the day of the biopsy. Boxplots show the median value, the mean and the standard error of the mean. Wilcoxon test revealed a statistical significance between groups with * *p* < 0.05; ** *p* < 0.01; *** *p* < 0.001 as indicated. TCMR = T-cell mediated rejection, AMR = antibody-mediated rejection.

In total, 65 biomarkers consisting of growth factors, chemokines and cytokines were mostly known from literature to be characteristic for the inflamed endothelium within rejection [4]. These were screened in plasma samples of 112 KTRs of the 1st cohort with 16 AMR-patients (Supplementary Table S9). In total, 14 markers high-scored by neural net analysis (Supplementary Tables S1 and S10) were selected and determined in plasma samples from the 78 KTRs in the 2nd patient cohort, including 23 patients with AMR. KTRs were defined according to BANFF-classification as ´rejection´ versus ‘non-rejection’ (Figure 2). CXCL9 was significantly increased in plasma samples of all patients with rejection and in the subgroup of KTRs with AMR, in addition in KTRs with borderline-TCMR of the 1st patient cohort (Figure 2A,B), whereas CXCL9 was not significantly elevated in the TCMR-defined-subgroup of both cohorts.

#### 3.2.2. Biopsy Lysate Screening

**Figure 3:**
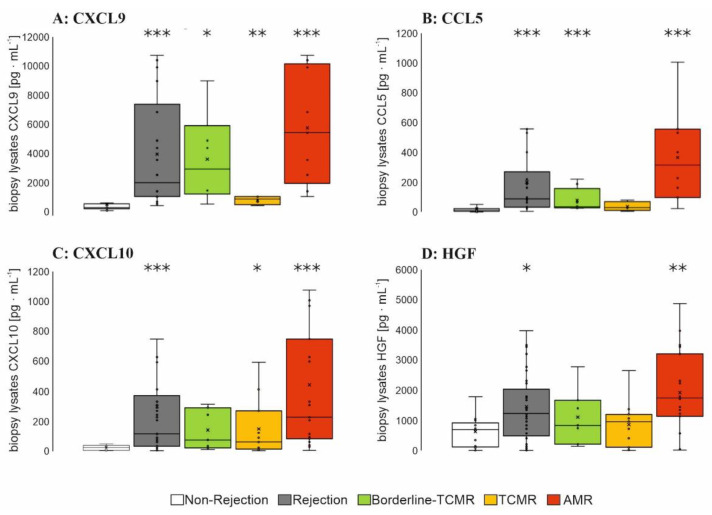
Multiplex ELISA analysis in biopsy lysates of the 1st patient cohort. Protein lysates were identified as inner cortex using microscopic analysis and grouped into non-rejection (*n* = 11), borderline-TCMR (*n* = 6), TCMR (T-cell mediated rejection, *n* = 9) and AMR (antibody-mediated rejection, *n* = 18). In total, 12.5 µg total protein was used to determine the concentrations of the cytokines/chemokines ((**A**) CXCL9, (**B**) CCL5, (**C**) CXCL10 and (**D**) HGF) using Luminex-based multiplex assay. Boxplots show the median value, the mean and the standard error of the mean. Wilcoxon test revealed a statistical significance between groups with * *p* < 0,05, ** *p* < 0.01, *** *p* < 0.001 as indicated.

To evaluate the presence in the renal cortex of the kidney graft, selected markers (Supplementary Table S11) were determined in 44 biopsy lysates of patients with a specific rejection form or with non-rejection. CXCL9 levels were increased in patients with AMR followed by patients with borderline-TCMR (Figure 3A). In parallel, levels of CCL5, CXCL10 and HGF (Figure 3B–D) were significantly higher in AMR, but not soluble CD25 (sCD25), CCL17 or thymic stromal lymphopoietin (TSLP) (Supplementary Table S10). CCL5 and CCL10 levels were also increased in biopsy lysates of borderline-TCMR-patients and TCMR-patients (Figure 3B–D).

#### 3.2.3. Urine Screening

**Figure 4:**
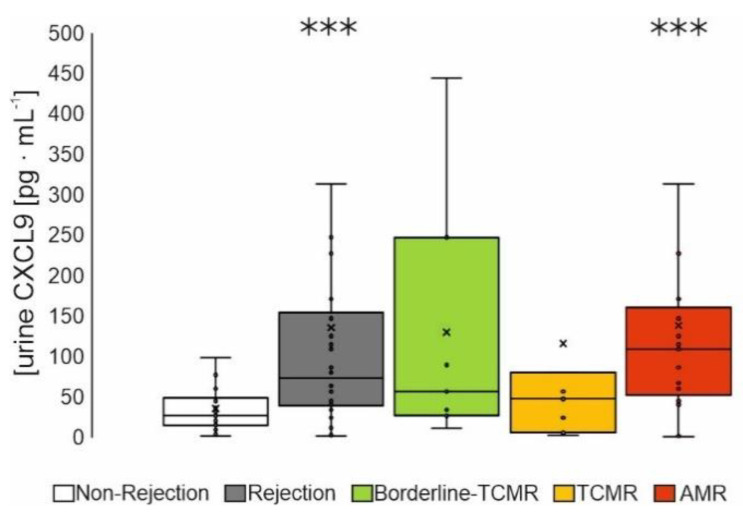
Urine samples of patients of the 2nd patient cohort were collected at the day of biopsy. Patients were grouped according to the histopathological scoring of their biopsy into patients with non-rejection (*n* = 40), borderline-TCMR (*n* = 8), TCMR (T-cell mediated rejection, *n* = 7) and AMR (antibody-mediated rejection, *n* = 23). Boxplots show the median value, the mean and the standard error of the mean. Wilcoxon test revealed a statistical significance between groups with *** *p* < 0.001 as indicated.

Luminex-based multiplex assay was performed within 78 whole-urine samples of the 2nd cohort using the 14 candidate biomarkers identified by neural network analysis (Supplementary Table S1). In accordance to results with plasma and biopsy lysates, CXCL9 levels were significantly increased in urine of AMR-patients (Figure 4) versus non-rejection, but not CXCL10 nor CCL5 and HGF (Supplementary Table S11).

### 3.3. CXCL9-Specific LFA

#### 3.3.1. Selection of a High-Affinity Monoclonal Antibody for the CXCL9-Specific LFA

Four antibodies with high affinity for CXCL9 were initially determined with the self-designed ELISA (Supplementary Figure S1A) and then analyzed for their affinity in low ranges of CXCL9 concentrations. Antibody A exhibited the highest affinity for CXCL9 (Supplementary Figure S1B) and was selected for the CXCL9-LFA.

#### 3.3.2. Coupling of a CXCL9-Binding Aptamer (AuNP-G123) to AuNPs

To determine CXCL9 in patient samples, conjugates of AuNPs and CXCL9-binding aptamer (G123) were prepared. If successful, conjugation of aptamers to the AuNP surface exhibited an increased diameter of nanoparticles without signs of agglomeration. The altered size of nanoparticles was verified using DLS and UV-vis (Supplementary Figure S2A,B). Here, the AuNP-G123 was compared to unmodified AuNPs exhibiting enlargement of particles due to the density distribution of particle diameter and the right shift of the wavelength. The functionality of the AuNP-G123 was verified via the LFA and non-specific binding partners were applied for a comparison (albumin, sCD25, CRP were used as negative controls; not shown).

#### 3.3.3. Design of the LFA and LOD

**Figure 5:**
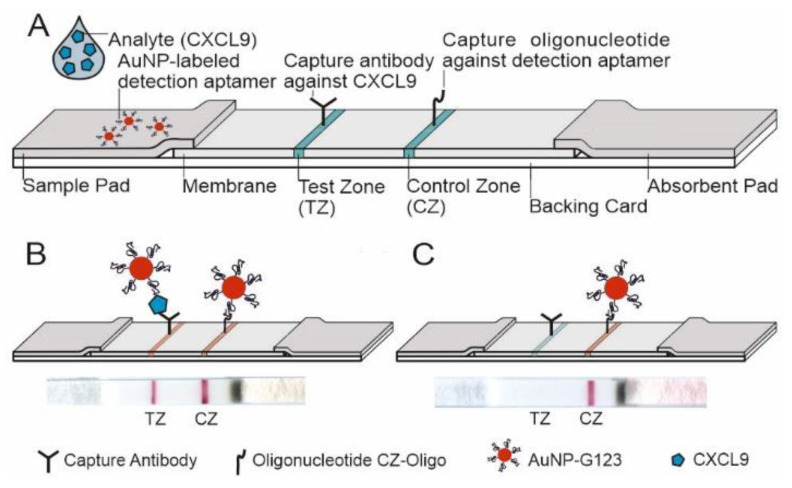
Schematic of the basic structure of the developed aptamer-antibody-hybrid-lateral flow assay (hybrid-LFA) with sample pad, nitrocellulose membrane and absorbent pad, providing the necessary lateral flow fluids and overlapping glued to a backing card. The hybrid-LFA uses a capture antibody in the test zone (TZ) and a detection aptamer conjugated to gold nanoparticles both with high affinity for CXCL9. (**A**) test platform composition, (**B**) positive test (two red lines, TZ and CZ), (**C**) negative test (one red line, CZ) (modified according to Seiler et al. [47]).

**Figure 6:**
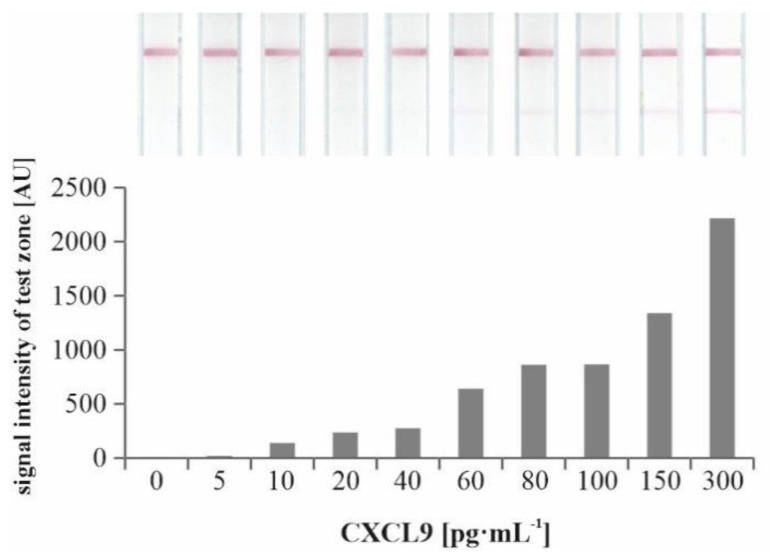
Lateral Flow Assays using different concentrations of purified CXCL9 in pooled urine samples of patients with unsuspicious finding proven by biopsy and binding buffer (1:1) with the corresponding columns reflecting the signal intensity of the test line. Scans were taken 45 min after sample application. N = 1.

**Figure 7:**
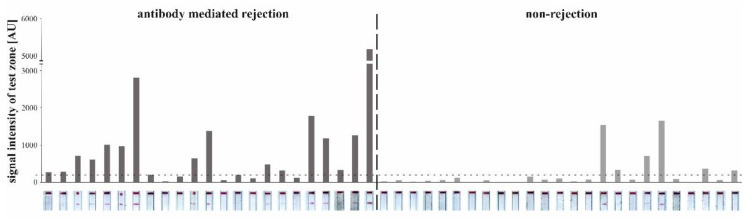
In total, 48 lateral flow assay strips tested with patient urine samples showing the test and control zone with the corresponding columns reflecting the signal intensity of the test line. Horizontal dashed line at 200 AU indicates the threshold between positive and negative test zone. N = 1.

Instead of conventionally applied paired antibodies, the presented LFA was designed as a sandwich assay with a capture antibody (named as antibody A, see Figure S1B) membrane-immobilized in the test zone and a detecting aptamer coupled to AuNPs (AuNP-G123). A short oligonucleotide sequence complementary to the aptamer was immobilized in the control zone (Figure 5A). CXCL9 in the sample applied binds to AuNP-G123, and this complex was finally fixed on the test zone by binding to the capture antibody. The marker line turns red and is visible if sufficient AuNP-G123-CXCL9 is bound (Figure 5B, Figure 6 and Figure 7). Without target CXCL9, no marker line appeared (Figure 5C, Figure 6 and Figure 7). When reaching the control zone, the oligonucleotide/AuNP-G123 complex forms a control line (Figure 5B,C) indicating that the test is functional (Figure 7).

To exclude non-specific LFA performance, a scrambled oligonucleotide exhibiting the same composition of bases as G123 but arranged in a different primary structure as well as unmodified AuNPs were used (Supplementary Figure S3). For determination of the limit of detection (LOD) of the LFA according to red color signals in the test zone, the system was exposed to technical samples using a serial dilution of purified CXCL9 in BB (0–300 pg·mL^−1^) and different running times (15 to 45 min). An LOD of 10 pg·mL^−1^ was determined with a running time of 45 min (Supplementary Figure S4). Spiked samples were prepared by using a 1:1 ratio of pooled urine samples of patients with unsuspicious finding proven by biopsy and binding buffer since pretests showed best results (Supplementary Figure S5). As compared to the test performance with technical samples, here an LOD of 60 pg·mL^−1^ CXCL9 was determined (Figure 6).

#### 3.3.4. Detection of CXCL9 by LFA in Patients with AMR

**Figure 8:**
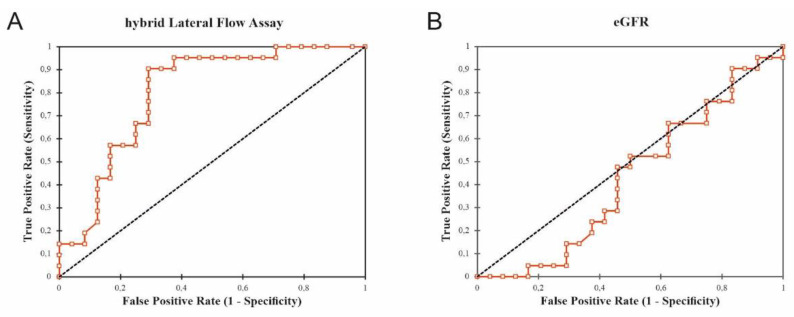
Area under the curve (AUC) values in receiver operating characteristic (ROC) analyses of (**A**) hybrid-LFA (AUC = 0.799) and (**B**) estimated glomerular filtration rate (eGFR, AUC = 0.428) at day of biopsy.

Evaluation of the developed LFA was performed in 48 urine samples of KTRs with AMR (23 samples) and non-rejection (25 samples). Scans after a running time of 15 min showed distinct red lines in the according zone for patient samples (Figure 7). LFAs with a signal intensity of the test zone lower than 200 AU according to ImageJ did not reveal a visible line in the test zone. Therefore, 200 AU was defined as threshold (horizontal dashed line, Figure 7). In the LFA presented in Figure 7, 17/23 AMR samples and 6/25 urine samples of patients with non-rejection showed a positive signal. LFA specificity and sensitivity was therefore determined to be 71% for each parameter. ROC analyses revealed that the AUC of the LFA was higher as compared to eGFR on the day of biopsy (LFA: AUC = 0.799 vs. eGFR: AUC = 0.428, 95% confidence interval, Figure 8). The positive predictive value of the new test was 0.652 and the negative predictive value was 0.708.

## 4. Discussion

Since AMR is a major risk for late transplant function loss [48,49], reliable and timely diagnosis making of AMR after Ktx is of major clinical importance. Some but not all patients with AMR exhibited increased serum creatinine, which is supported by a recent molecular in-depth analysis of indication biopsies [50] that frequently turns out to be acute kidney injury and/or chronic kidney disease rather than transplant rejection in patients with low eGFR. Allo-immunological risk criteria such as high PRA percentage and presence of detectable DSAs were not fulfilled for patients with AMR of this work. PRA was highest in patients with non-rejection following not predictive for AMR. Defined DSAs were detectable in some but not all AMR-patients and various patients with borderline-TCMR indicating the ambiguous nature of these diagnostic tools.

The aim of this work was to identify potentially suitable immunological biomarkers in plasma that may reveal AMR in an early stage of this condition. To this end, a set of 65 parameters including cytokines and chemokines [4,51] were determined in a first patient cohort and best scored markers according to neural net analysis were comparatively tested for statistical significance in AMR among other biopsy-specific subgroups in the 2nd patient cohort using a thesis-driven statistical test (Wilcoxon analysis). In plasma samples, CXCL9 was identified as significant marker for AMR despite effective immunosuppression drug levels, which may confirm, that chemokine production is at least partially refractory towards systemic immunosuppression [42]. The requirements for a diagnostic for AMR are quite high; it must perform for many patients with very different preconditions with regard to their immune system, pre-immunization and medication. Despite that, we were able to select the best possible indicator for AMR within the marker panel selected.

CXCL9 has previously been shown to have diagnostic potential as urine biomarker within TCMR [52,53,54,55,56,57] and BK virus nephropathy together with CXCL10 [58]. Compared to serum creatinine, CXCL9 indicates graft rejection earlier and more sensitively [3,51,59]. We could show that CXCL9 is highly elevated in urine of KTRs with AMR, which is in line with results of others [54]. Of note, the CXCL9 mean level was higher in AMR than in TCMR urine samples and CXCL9 was not significantly increased in serum and urine of TCMR patients of the cohorts reported here. This is contrary to earlier publications and could be due to the fact that the percentage of TCMR in the biopsy findings of the cohorts presented here was relatively low (due to a very close monitoring of KTRs). TCMR and chronic AMR in patients of the 2nd cohort was detected relatively late, which is characteristic for long-term transplanted patients in the weaning-phase of immunosuppression, justified by an increasing rate of immunosuppression-induced side effects. Furthermore, some patients of the 2nd cohort were not so closely monitored by protocol biopsies, which is obvious according to a decreased percentage of protocol than indication biopsies in this group (Supplementary Table S2). Furthermore, we cannot confirm the positive indicative character of CXCL9 for TCMR with this new POCT in general, and also its value for borderline-TCMR would have to be evaluated further.

CXCL9, mainly produced by human macrophages in response to interferon-γ [60,61,62,63], has also been shown to be produced in activated renal graft endothelium [59], which was confirmed by comparative analysis of renal cortex lysates from AMR-patients to those of patients without rejection in this work: here CCR3 ligands CXCL9 as well as CXCL10 were increased within AMR [42,51], suggesting a regulatory role for plasma and NK cells recruitment [59], accompanied by increased levels of the heat-shock-protein-regulated chemokine CCL5 [64] and hepatocyte growth factor (=HGF) [51], associated with increased mitogenic and morphogenic activities in endothelial cells [65]. In addition, a low CXCL10/creatinine ratio—determined in urine—was also successfully used for prediction of rejection free episodes during the first year after kidney transplantation [66].

As limitation of the work presented here is the fact, that despite a positive screening for the presence of HLA I-or II-DSAs, not in all patient samples tested with the hybrid-LFA, single antigen HLA-DSAs could be identified. Non-HLA-antibody screening was not available at that time. At the time of recruiting the 1st cohort, the presence of DSA was not yet an obligatory criterion for the diagnosis of chronic AMR, whereas it was postulated for this diagnosis according to the BANFF Classification 2015 [5,40] for patients of the 2nd cohort. This leads to the statement, that some of the patients in the 2nd cohort were only “highly suspicious for AMR” and further clinical studies have to be performed with this assay.

Alternative biomarker or tests for these cases are highly warranted and qualitatively differing assays have recently been evaluated. However, neither autoantibodies caused by organ-derived autoantigens after surgical trauma or transplant ischemia [67,68] nor cell-free DNA from dying cells in a rejected renal transplant [69] have been evaluated for AMR screening after Ktx.

Since in this work, CXCL10 was only significantly increased at the time of chronic AMR diagnosis in biopsy lysates (Figure 3C), but not in urine (Supplementary Table S11), we developed a CXCL9-focused assay. Here, a CXCL9 specific LFA was developed for a urine POCT in AMR. To this end, antibody evaluation by sandwich testing specified 1 out of 19 rat monoclonal antibodies with high target-specificity and capture capacity for CXCL9. Using these affine biomolecules, a hybrid-LFA setup was designed as a binary diagnostic tool for self-monitoring in patients´ urine after Ktx leading to a sensitivity and specificity of 71%, respectively. In parallel, a traditional solely antibody-based LFA yielded only a sensitivity of 53% with comparable specificity of 71% [70]. We suggest that the relatively smaller size of the 82 bp aptamer (size approximately 26.2 kDa [71]) could be a key point for the improved sensitivity especially with respect to the small target protein CXCL9 (11.7 kDa [72]). A second antibody as coupling partner on the nanoparticles as detection molecule could also impose a relevant steric hindrance due to its size (mean size of an IgG-antibody: ~150 kDs), possibly not offering the optimal binding pocket for the small CXCL9 molecule. The smaller aptamer tends to have better access to CXCL9, esp. if CXCL9 is still bound to the scavenger. Therefore, the combination of antibody and aptamer at the appropriate binding sites proves to be more efficient for CXCL9 sandwich binding. The hybrid-LFA presented here could be especially helpful in KTRs of risk, e.g., with a high pre-immunization score or with newly identified donor specific antibodies, to screen for subclinical AMR. This new aptamer–antibody-hybrid-LFA shows a higher AUC according to ROC analysis for AMR than eGFR (AUC of 0.799 as compared to 0.428) at the day of biopsy.

Despite the promising findings in AMR-patients or patients with a kidney graft highly suspicious for AMR, further studies are required to narrow the value of this hybrid-LFA. Its suitability should also be clearly differentiated against non-rejection-related immunological diseases such as a systemic inflammation or a specific graft or urogenital infection. In addition, studies using such an easy-to-use-CXCL9-POCT could clarify the precise onset of CXCL9 production within AMR in correlation with biopsy findings.

## Figures and Tables

**Table 1:** Summary of used ssDNAs and their characterization (all obtained from ELLA Biotech, Planegg, Germany).

Name	Sequence	Modification	Target
G123	CACGACGCAAGGGACCACAGGGAGGGAGGGTGGGCAAAGGGCCCTAAGTCCGTAACAAAAACACAGCACGACACCGCAGAGGCA	C6-SS-HEG-5′-3′	CXCL9
CZ-Oligonucleotide	TGCCTCTGCGGTGT CGTGCT	biotin-HEG-HEG-5′-3′	G123
Control-Oligonucleotide	CACGACGCAAGGGACCACAGGAGGAGAGTAGGCGATACACGACGTAGCGCAGATAGGACCAAGCAGCACGACACCGCAGAGGCA	C6-SS-HEG-5′-3′	

## Data Availability

Not applicable.

## Supplementary Materials

The following are available online at www.mdpi.com/article/10.3390/diagnostics12020308/s1, Figure S1: ELISA results with supernatants of hybridoma produced in rats; Figure S2: Comparison of gold nanoparticle-aptamer conjugates (AuNP-G123) and unmodified gold nanoparticles (AuNP); Figure S3: Comparison of three different AuNP-conjugates to exclude non-specific binding; Figure S4: Lateral Flow Assays using different concentrations of CXCL9 in binding buffer; Figure S5: Signal intensity of the test zone of lateral flow assays using five different patient sample-to-binding buffer-ratios (10%, 25%, 50%, 75% and 100% patient sample with antibody-mediated rejection); Table S1: List of immunological parameters screened in plasma and urine samples of the 2nd cohort; Table S2: General information about the kidney transplant recipients and their biopsies; Table S3: Patients’ characteristics, 1st patient cohort; Table S4: Patients’ characteristics, 2nd patient cohort (16 patients, including 12 children); Table S5: Characteristics, course and donor-specific HLA (human leucocyte antigen)-antibodies in patients (1st cohort) with antibody-mediated rejection in the follow up of 4.5 years; Table S6: Characteristics and donor-specific HLA (human leucocyte antigen) -antibodies in patients (2nd cohort) with antibody-mediated rejection in the follow up of 9 months; Table S7: Serological tests 1st patient cohort. Table S8: Serological tests 2nd patient cohort; Table S9: List of all immunological parameters screened in plasma samples of the 1st cohort; Table S10: Continuous scaled variables: discriminators for rejection (borderline-T-cell mediated rejection, T-cell mediated rejection, antibody-mediated rejection) or unsuspicious state (non-rejection); Table S11: List of all immunological parameters screened in biopsy lysates of the 1st cohort.
