# Supplementary material for: An Antibody-Aptamer-Hybrid Lateral Flow Assay for Detection of CXCL9 in Antibody-Mediated Rejection after Kidney Transplantation
Source: Diagnostics (Basel). 2022 Jan 25;12(2):308. doi: 10.3390/diagnostics12020308 (PMC8871475; doi:10.3390/diagnostics12020308)
Supplement: Supplementary file 1 [file diagnostics-12-00308-s001.zip › diagnostics-1515174-supplementary.pdf]

Supplementary Materials

Tables

Table S1: List of immunological parameters screened in plasma and urine samples of the 2nd cohort.

	Immunological parameter
1	GM-CSF
2	MIF
3	CXCL9
4	CCL17
5	CCL26
6	HGF
7	IFN-a2
8	sCD25
9	IL-13
10	IL-15
11	PDGF-BB
12	CCL5
13	TNF-b
14	TSLP

Table S2: General information about the kidney transplant recipients and their biopsies. TCMR = T-cell mediated rejection, AMR = antibody-mediated rejection.

	1st patient cohort			2nd patient cohort	
Variable	n / N	%	Rejection in Follow Up biopsies	n / N	%
Female	48 / 112	42.9		28 / 78	35.9
Male	64 / 112	57.1		50 / 78	64.1
Protocol biopsy	62 / 112	55.4		27 / 78	34.6
For-cause-biopsy	50 / 112	44.6		51 / 78	65.4
Non-rejection	52 / 112	46.4	4 x TCMR,<br>2 x borderline-TCMR	40 / 78	51.3
Borderline-TCMR	31 / 112	27.7	4x borderline-TCMR,<br>2 x AMR,<br>2 x AMR / TCMR,<br>3 x TCMR	8 / 78	10.3
TCMR	13 / 112	11.6	3 x TCMR,<br>1 x AMR / TCMR,<br>1 x borderline-TCMR	7 / 78	8.9
AMR	16 / 112	14.3	9 x AMR	23 / 78	29.5

Table S3: Patients´ characteristics, 1st patient cohort.

	Non-rejection	TCMR	Borderline-TCMR	AMR
Females	18 / 52	4 / 13	16 / 31	4 / 16
Age	48.0 ± 14.9	54.5 ± 18.5	52.8 ± 14.3	49.0 ± 12.2
Transplant age [months]	25.4 ± 55.7	11.5 ± 20.7	23.2 ± 55.6	64.3 ± 75.7
Primary non function	6 / 52	4 / 13	5 / 31	1 / 16
Immunosuppressive drug regimen	19 CyA*,<br>33 Tac*	5 CyA*,<br>8 Tac*	12 Tac*,<br>12 Cya*,<br>2 Cya / steroid-free,<br>2 mTOR-MPA,<br>2 mTOR*,<br>1 CyA / Aza steroid free	3 CyA*,<br>7 Tac*,<br>2 Tac dual,<br>2 Cya dual,<br>1 Cya mono,<br>1 Steroid mono
Living donations	22 / 52	2 / 13	10 / 31	3 / 16
Previous rejections	16 / 52	3 / 13	17 / 31	8 / 16
Re-graft	9 / 52	0 / 13	5 / 31	4 / 16

Table S4: Patients´ characteristics, 2nd patient cohort (16 patients, including 12 children).

	Non-rejection	TCMR	Borderline-TCMR	AMR
Females	22 / 40	3 / 7	4 / 8	8 / 23
Age	59 ± 2.8	43.45 ± 20.3	40.4 ± 23.1	38.9 ± 22.8
Transplant age [months]	110 ± 110.3	94 ± 98.7	71.2 ± 100.6	130.1 ± 103.9
Primary non function*	3/40	no	no	no
Immunosuppressive drug regimen	25 Tac*,<br>9 mTOR<br>3 CyA/MPA,<br>3 CyA-Aza-steroids	4 Tac*,<br>1 Cya*/MPA,<br>2 mTOR*	3 Tac*,<br>3 mTOR*<br>2 mTOR-steroids	10 Tac*,<br>3 mTOR*,<br>6 mTOR-CI,<br>1 CI-Aza-steroids,<br>3 CI steroid-free
Living donations	8 / 40	4 / 7	2 / 8	9 / 23
Previous rejections	No	no	no	8
Re-graft	No	no	no	3

*Primary non function was defined by the necessity of more than one dialysis treatment after kidney transplantation

<u>Legend Table S3 and Table S4:</u>

CyA* = standard triple therapy comprising cyclosporine, mycophenolate and steroids
Tac* = standard triple therapy comprising tacrolimus, mycophenolate and steroids
mTOR* = standard triple therapy comprising mTOR-inhibitor, mycophenolate and steroids
CyA dual = cyclosporine combined with steroids
Tac dual = tacrolimus combined with steroids
CI steroid-free = calcineurin inhibitor combined with mycophenolate
mTOR-steroids = mTOR-inhibitor combined with steroids
mTOR-CI-steroids = mTOR-inhibitor combined with CI and steroids
mTOR-CI-MPA = mTOR-inhibitor combined with CI and mycophenolate
mTOR-CI = mTOR-inhibitor combined with CI without steroids
CI-Aza-steroids = calcineurin inhibitor combined with azathioprine and steroids
CI-Aza = calcineurin inhibitor combined with Azathioprine
Cya mono = limited immunsuppression with cyclosporine only
Steroid mono = limited immunosuppression with steroids only
TCMR = T-cell mediated rejection
AMR = antibody-mediated rejection

Table S5: Characteristics, course and donor-specific HLA (human leucocyte antigen)-antibodies in patients (1st cohort) with antibody-mediated rejection in the follow up of 4.5 years. DSA=donor specific antibody.

	Patient age [years]	Transplant age [months]	Specificity of DSAs	Transplant loss within 4.5 years	Ongoing rejection / further episodes
1	41	28	DQ6	Yes	AMR
2	50	145	DQ7	Yes	AMR
3	41	43	-	No	No
4	51	22	-	No	No
5	54	145	DQ7	Yes	AMR
6	47	249	A1 (30,31,33)	No	No
7	50	12	DPB0501	Yes	AMR
8	64	4	-	Yes	No
9	21	65	-	Yes	AMR
19	40	3	-	No	No
11	50	1	DQ9	No	AMR
12	41	36	-	Yes	AMR
13	50	70	-	No	No
14	75	0	-	No	No
15	51	38	-	Yes	AMR
16	37	98	-	No	AMR
Σ			6 / 16	8 / 16	9 / 16

Table S6: Characteristics and donor-specific HLA (human leucocyte antigen) or Major-histocompatibility-complex (MHC) class I–related chain A (MICA) antigen-antibodies in patients (2nd cohort) with antibody-mediated rejection in the follow up of 9 months.

	Patient age [years]	Transplant age [months]	Single defined donor specific HLA antibodies	Ongoing rejection / further episodes
1	44	228	DRB1	AMR
2	20	216	DQ 4, 5, 6	AMR
3	81	122	DQA1	AMR
4	34	284		AMR
5	41	202		AMR
6	44	396	DQ5	AMR
7	118	340		AMR
8	34	44		AMR
9	71	112	A1	AMR
19	73	98		AMR
11	39	352		Tx glomerulopathy
12	57	155	DQB1	AMR
13	26	85		AMR
14	72	44		AMR
15	23	82		chronic TCMR
16	54	225		AMR
17	23	62	DRB1	AMR
18	40	280	DQ7	AMR
19	53	55	DQA1	AMR
20	38	30		AMR
21	35	254		AMR
22	64	55	DQA1	AMR
23	43	254	DQ5	AMR
Σ			11 / 23	23 / 23

Table S7: Serological tests 1st patient cohort. TCMR=T-cell mediated rejection, AMR=antibody-mediated rejection.

	Non-rejection	TCMR	Borderline-TCMR	AMR
PRA (%)	12 patients, range: 6-100 %, median 76 %	no patient	6 patients, range: 8-25 % median 22 %	8 patients, 2 - 70 %, median 39 %
HLA I	no patient	no patient	3 patients	12 patients, see table S5
HLA II	12 patients	1 patient	6 patients	
Single antigen test	no patient	no patient	1 patient	

Table S8: Serological tests 2nd patient cohort. TCMR=T-cell mediated rejection, AMR=antibody-mediated rejection.

	Non-rejection	TCMR	Borderline-TCMR	AMR, see table S6
PRA (%)	16 patients range: 7-100% median: 56%	4 patients; Range: 62-100%; median: 50%	3 patients; range: 74-89% median: 89%	23 patients range: 0-100% median: 23%
HLA I	no patient	2 patients	no patient	5 patients
HLA II	5 patients	2 patients	1 patient	18 patients
Single antigen test	1 patient	no patient	1 patient	11 patients

Table S9: List of all immunological parameters screened in plasma samples of the 1st cohort.

	Immunological parameter		Immunological parameter		Immunological parameter
1	IL-1a	23	TRAIL	45	CCL2
2	sCD25	24	VCAM-1	46	CCL3
3	IL-3	25	IL-1b	47	PDGF-bb
4	IL-12	26	IL1RA	48	CCL4
5	p40	27	IL-2	49	CCL5
6	IL-16	28	IL-4	50	TNF-a
7	IL-18	29	IL-5	51	VEGF
8	CCL27	30	IL-6	52	CLL24
9	CXCL1	31	IL-7	53	CXCL13
10	HGF	32	CXCL8	54	CCL13
11	ICAM-1	33	IL-9	55	CCL17
12	IFN-a2	34	IL-10	56	CCL21
13	LIF	35	IL-12p70	57	CCL26
14	CCL7	36	IL-13	58	TPO
15	M-CSF	37	IL-15	59	TSLP
16	MIF	38	IL-17	60	CXCL5
17	CXCL9	39	CCL11	61	CCL15
18	b-NGF	40	FGF b	62	sCD31
19	SCF	41	G-CSF	63	Leptin
20	SCGF-b	42	GM-CSF	64	Ang-2
21	CXCL12	43	IFN-g	65	Follistatin
22	TNF-b	44	CXCL10		

Table S10: Continuous scaled variables: discriminators for rejection (borderline-T-cell mediated rejection, T-cell mediated rejection, antibody-mediated rejection) or unsuspicious state (non-rejection).

variable	Mean ± sd; rejection versus non-rejection	Score	Significance according to Wilcoxon-Test
GM-CSF ($pg \cdot ml^{-1}$)	5.1 ± 14.7 vs. 10.8 ± 22.7	0.9	Ns
Serum Creatinine at biopsy ($\mu M \cdot l^{-1}$)	256.8 ± 93.2 vs. 170.8 ± 80.9	0.83	<0.001
CXCL9 ($pg \cdot ml^{-1}$)	1586.0 ± 269.3 vs. 818.8 ± 152.2	0.79	<0.03
IL-13 ($pg \cdot ml^{-1}$)	14.5 ± 12.0 vs.12.9 ± 8.6	0.73	Ns
MIF ($pg \cdot ml^{-1}$)	797.4 ± 757.1 vs. 413.9 ± 212.1	0.61	Ns
IFN-2α ($pg \cdot ml^{-1}$)	39.8 ± 16.4 vs. 45.0 ± 15.3	0.54	Ns
IL-15 ($pg \cdot ml^{-1}$)	9.0 ± 5.7 vs. 9.0 ± 6.0	0.54	Ns
sCD25 ($pg \cdot ml^{-1}$)	126.4 ± 98.6 vs. 363.6 ± 216.02	0.54	<0.05
CCL17 ($pg \cdot ml^{-1}$)	11.5 ± 7.8 vs. 16.5 ± 16.9	0.53	Ns
CCL5 ($pg \cdot ml^{-1}$)	833.1 ± 447.7 vs. 692.5 ± 300,0	0.52	Ns
CCL26 ($pg \cdot ml^{-1}$)	20.6 ± 10.8 vs. 20.9 ± 22.7	0.52	Ns
TSLP ($pg \cdot ml^{-1}$)	1.7 ± 0.7 vs. 2.5 ± 7.2	0.51	0.01
HGF ($pg \cdot ml^{-1}$)	145.7 ± 110.4 vs. 161.4 ± 100.1	0.50	Ns
TNF-α ($pg \cdot ml^{-1}$)	0.5 ± 0.7 vs. 0.7 ± 0.9	0.49	Ns
PDFG-bb ($pg \cdot ml^{-1}$)	271.7 ± 471.5 vs. 454.2 ± 667.5	0.48	<0.08

Table S11: List of all immunological parameters screened in biopsy lysates of the 1st cohort.

	Immunological parameter		Immunological parameter		Immunological parameter
1	IL-1a	18	SCF	35	IL-13
2	sCD25	19	SCGF-b	36	IL-15
3	IL-3	20	CXCL12	37	IL-17
4	IL-12 p40	21	TNF-b	38	CCL11
5	IL-16	22	TRAIL	39	FGF b
6	IL-18	23	VCAM-1	40	G-CSF
7	CCL27	24	IL-1b	41	GM-CSF
8	CXCL1	25	IL1RA	42	IFN-g
9	HGF	26	IL-2	43	CXCL10
10	ICAM-1	27	IL-4	44	CCL2
11	IFN-a2	28	IL-5	45	CCL3
12	LIF,	29	IL-6	46	PDGF-bb
13	CCL7	30	IL-7	47	CCL4
14	M-CSF	31	CXCL8	48	CCL5
15	MIF	32	IL-9	49	TNF-a
16	CXCL9	33	IL-10	50	VEGF
17	b-NGF	34	IL-12p70		

Table S11: Comparison of other biomarker levels, detected in urine of patients with antibody-mediated rejection (AMR) or no rejection.

variable	Mean ± sd AMR versus no rejection	Significance according to Wilcoxon-Test
CXCL10 (pg/mL)	89.33±105.22 vs. 37.2±31.2	ns
CCL5 (pg/mL)	143.87± 457.34 vs. 6.44 ± 4.37	ns
HGF (pg/mL)	66.42 ± 44.62 vs. 65.88 ± 71.93	ns

Figures

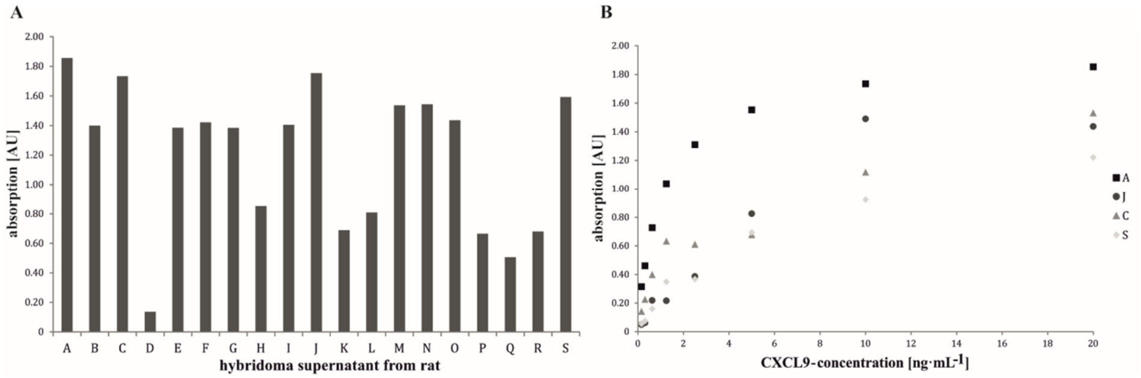

Figure S1: ELISA results with supernatants of hybridoma produced in rats. A) OD of antibodies in 19 different hybridoma supernatants (A-S) B) Comparison of the four best capture antibodies (A, J, C, S) from A.

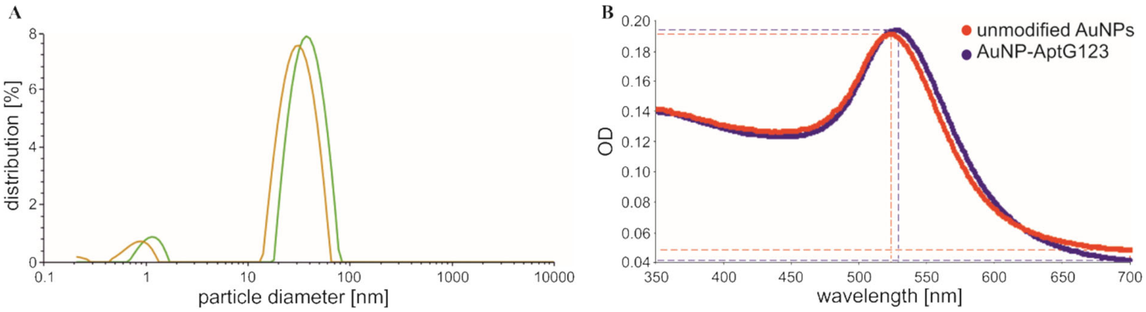

Figure S2: Comparison of gold nanoparticle-aptamer conjugates (AuNP-G123) and unmodified gold nanoparticles (AuNP). Preparation of AuNP-aptamer conjugates (AuNP-G123) was performed according to the protocol of Phung et al.[43], here AuNP-aptamer mixture was incubated in 20 nM citrate buffer overnight, AuNP-aptamer conjugation mix was washed twice with 1% sucrose and settings for centrifugation (6000 xg, 30 min, 20°C) were adjusted. A) Particle size distribution of diluted unmodified gold nanoparticles (orange graph) and gold nanoparticle-aptamer conjugate (AuNP-G123, green graph, diluted 1:3000 in water) using dynamic light scattering. B) Characterization of unmodified AuNP (red graph) and AuNP-G123 (blue graph, diluted 1:5 in 1% sucrose) using UV-Vis.

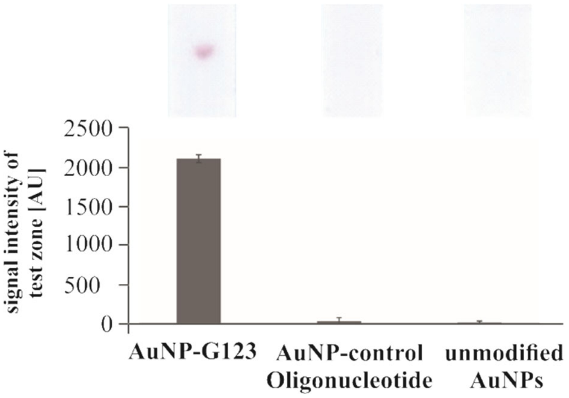

Figure S3: Comparison of three different AuNP-conjugates to exclude non-specific binding. Lateral flow assays were run with 1 ng·mL^{-1} CXCL9. N=3.

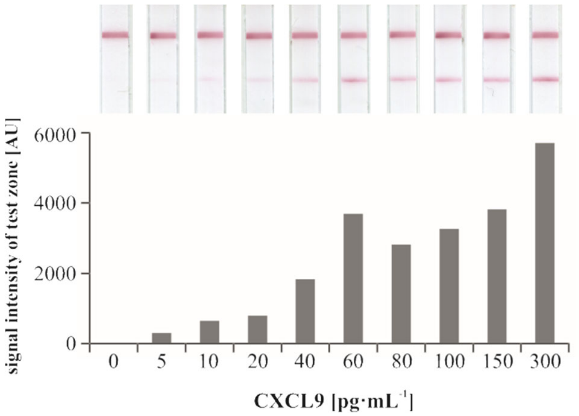

Figure S4: Lateral Flow Assays using different concentrations of CXCL9 in binding buffer. Scan was taken 45 minutes after sample application. N=1.

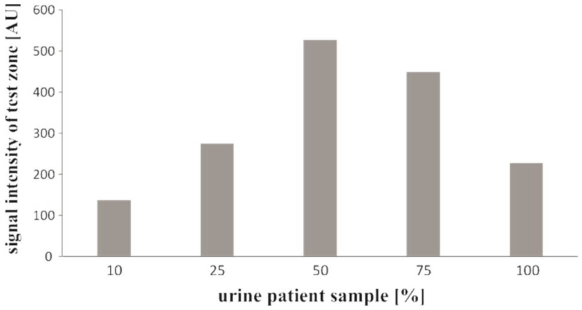

Figure S5: Signal intensity of the test zone of lateral flow assays using five different patient sample-to-binding buffer-ratios (10%, 25%, 50%, 75% and 100% patient sample with antibody-mediated rejection). N=1.
